# Grey water footprint of pharmaceuticals and personal care products discharged via urban wastewater

**DOI:** 10.1038/s41598-026-48905-5

**Published:** 2026-04-17

**Authors:** Lada Stejskalová, Libor Ansorge, Pavel Rosendorf, Daniel Fiala, Yelizaveta Chernysh, Angeles Blanco, Laetitia Minguez, Jiří Kučera, Anna Břicháčková, Dagmar Vološinová, Lenka Smetanová, Miroslav Váňa

**Affiliations:** 1 public research institution, T. G. Masaryk Water Research Institute, Podbabská 2582/30, 16000 Prague, Czech Republic; 2 International Innovation and Applied Center “Aquatic Artery”, Sumy State University, Kharkivska st. 116, Sumy, 40007 Ukraine; 3 Environmental Problems Research and Application Center, Muğla Sıtkı Koçman University, Kötekli Yerleşkesi, 48050 Menteşe, Muğla Turkey; 4 Department of Sustainable Technologies, Faculty of Tropical AgriSciences, Czech University of Life Sciences Prague, Kamýcká 129, 16500 Prague, Czech Republic; 5 Complutense University of Madrid, 28015 Madrid, Spain; 6 University of Lorraine, CNRS, LIEC, 57000 Metz, France

**Keywords:** Grey water footprint, Micropollutants, Pollutant priorization, PPCP, Urban wastewater, Water pollution, Environmental sciences, Hydrology, Water resources

## Abstract

Urban wastewater is a significant source of micro- and macropollutants in aquatic ecosystems, posing a high potential risk to drinking water sources. Pharmaceuticals and personal care products (PPCPs) are widely recognized as contaminants of emerging concern, although their relative burden compared to traditional pollutants remains insufficiently quantified. This study presents a comprehensive assessment of 92 micropollutants together with conventional pollution represented by nutrients and organic pollutants across 19 municipalities in the catchment of the largest Czech drinking water reservoir. More than one hundred 24-hour composite measurements were analysed. The Grey Water Footprint methodology integrates ecotoxicological thresholds, enabling a consistent comparison across PPCPs, nutrients, and organic pollution. Results reveal that the most critical micropollutants released from centralized wastewater treatment plants are Ibuprofen or Diclofenac, depending on treatment technology and plant size. However, nitrogen remains the dominant stressor approximately one kilometer downstream of urban discharges. These findings highlight that despite the increasing attention to micropollutants, conventional pollutants still account for the largest share of pollution in recipients. The methodological framework applied in this study allows stakeholders to compare the risks of different types of pollutants in a specific region. It offers a transferable tool for prioritizing contaminants and treatments, and guiding local wastewater management strategies under the EU Water Framework Directive, risk assessments under the revised Urban Wastewater Treatment Directive, and beyond.

**Supplementary Information:**

The online version contains supplementary material available at 10.1038/s41598-026-48905-5.

## Introduction

Ensuring high-quality drinking water is a critical global challenge, encompassing various factors such as water management policies, technological advancements, climate change and environmental impact, population growth and urbanization, agricultural practices, public awareness, and economic investments.

The most significant threats to water quality in drinking water reservoirs include diffuse pesticide sources upstream of the reservoir, and pharmaceuticals and personal care products (PPCPs) released from urban wastewater treatment plants (WWTPs)^1^. According to the best available data to date, and recognizing uncertainties in the data gathered, pharmaceuticals represent 59% of input quantities of micropollutants to urban WWTPs, and personal care products contribute 14%. From a different perspective, pharmaceuticals represent 48% of the toxic chronic load and personal care products contribute 17%^2^. These emerging contaminants (EC) can endanger the population through both acute and chronic exposure^3,4^. Urban WWTP discharges are generally regarded as dominant sources of EC, whereas discharges from manufacturing, hospitals, animal husbandry, and aquaculture can gain importance as local hotspots^5^. A key question is how to assess the impact of exposure to these mixtures. Although much research has been done on this topic, the need for empirical data to improve risk assessments has recently been identified as a key factor^3^.

Considering the degree of freshwater pollution and the ecotoxicological effects, the grey water footprint (GWF) indicator, derived from the “water footprint“ concept introduced in 2002^6^, is one of the most appropriate tools to compare anthropogenic impacts using a holistic approach^7,8^. It is defined as the volume of freshwater required to assimilate a load of pollutants, taking into account their natural background concentrations and existing ambient water quality standards^9^. Although it has certain conceptual and methodological shortcomings^10,11^, its strength lies in its ability to interpret usually complex situations. GWF has been mainly used to assess standard chemical parameters related to organic and nutrient pollution^12–17^. However, it can also be applied to micropollutants, although only a few studies have addressed this application. Martínez-Alcalá et al.^18^ studied persistent pharmaceuticals (Carbamazepine, Diclofenac, Ketoprofen, and Naproxen) entering ecosystems via wastewater discharges. Wöhler et al.^19^ assessed water pollution caused by human and veterinary pharmaceuticals, considering three geographical levels: global, national (of Germany and the Netherlands), and watershed level. They also derived an integrated approach to assess the GWF of veterinary antibiotics^20^. Other studies include a master’s thesis focusing on the GWF of antibiotics used in intensive aquaculture production^21^; a study by Ansorge et al.^22^ focusing on the GWF of EC originating from urban wastewater in the Sava River Basin; an assessment of EC as part of the Joint Danube Survey^23^; and a GWF case study at the Bandung WWTP in Indonesia^24^. Recently, a combined assessment of the GWF of nutrients and pesticides in a lake basin in sub-Saharan Africa has been carried out^25^. With the exception of studies^17,18,24,25,32^, the existing literature largely lacks comparisons of the pollution burden caused by micropollutants and macropollutants. These studies provided evidence that, in some cases, EC may pose a greater environmental risk than commonly monitored macropollutants. Therefore, the current state of the art demonstrates the need to include micropollutants in GWF studies.

This study represents the first comprehensive assessment, rigorously quantifying and comparing the environmental burden caused by PPCPs with nutrient and organic pollutants discharged via urban wastewater; evaluates their ability to overcome conventional or non-conventional wastewater treatments; and examines their ability to spread in surface waters. Applying the GWF methodology to a robust dataset, along with the included sensitivity analysis addressing the variability in pollution threshold values, addresses a significant scientific gap regarding the impact of urban wastewater discharges.

The case study was carried out in the watershed of the Švihov Water Reservoir (built on the Želivka River), the largest drinking water reservoir in Central Europe, spanning an area of 16 km^2^ (water abstraction coordinates 49.7247244 N, 15.0908753E).

## Materials and methods

### Monitoring

Švihov Water Reservoir was selected because it represents one of the critical infrastructures of the Czech Republic, in operation for more than 40 years, providing drinking water to more than 10% of the population of the Czech Republic (the capital city of Prague and a large part of the Central and South Bohemian regions).

**Scheme 1:**
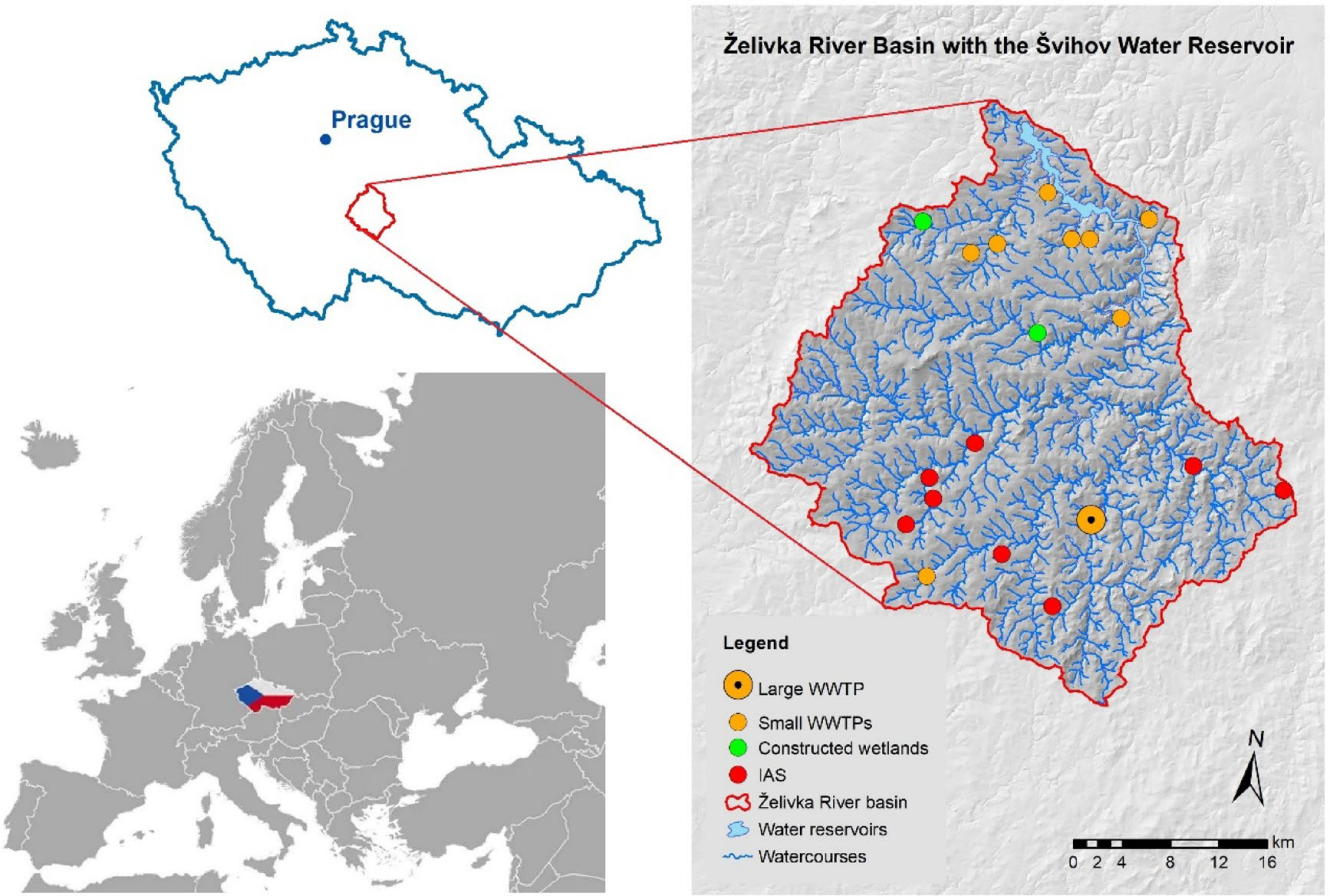
Map of the study area – the Želivka River Basin, Czech Republic. Created using ArcGIS Desktop 10.8.2 (https://www.esri.com).

Based on previous monitoring efforts (e.g.^26^ in the Švihov water reservoir watershed - nineteen representative municipalities/urban pollution sources were selected for this study (Scheme 1):

- Eight small municipalities (from 60 to 230 inhabitants), without a central WWTP, where wastewater is addressed via Individual Appropriate Systems (IAS). Wastewater is treated to varying degrees, often only using old septic tanks with discharge into a combined sewer (which originally served as a storm sewer) and subsequent combined discharge into the recipient. Pollution removal usually occurs under anaerobic conditions. which does not provide the conditions for the removal of some types of pollution, such as the oxidation of ammonia nitrogen. Another important mechanism for reducing concentrations is the dilution of wastewater with ballast water (leakage into the sewer). The average effluent flow is 0.9 L.s^−1^ (SD 1.0; median 0.5; max 4.4; min 0.04).
- Two small municipalities (120 to 200 inhabitants) with a central unconventional WWTP - constructed wetlands. In treatment beds with horizontal flow, anaerobic conditions typically occur. The average effluent flow is 1.2 L.s^−1^ (SD 0.6; median 1.3; max 2.0; min 0.3).
- Eight small municipalities (80 to 960 inhabitants) equipped with a central conventional WWTP - mechanical-biological technology with activated sludge process. In the Czech Republic, even small WWTPs are designed with a high sludge age so that activated sludge is able to efficiently remove ammonia nitrogen, and possibly total nitrogen (through the denitrification process). Phosphorus precipitation is often also included, which reduces effluent phosphorus concentrations, but also affects the properties of the activated sludge. The average efluent is 1.3 L.s^−1^ (SD 1.3; median 0.7; max 5.9; min 0.1).
- One medium-sized municipality (15,400 inhabitants) equipped with a large WWTP employing a mechanical-biological treatment with a low-load activated sludge process, achieving the highest removal efficiencies. To protect the water reservoir, low effluent concentration limits for nitrogen (14 mg.L^−1^) and phosphorus (1 mg.L^−1^) are required. Although categorized as a medium-sized facility (36,000 PE), this WWTP is referred to as ‘large’ in this study to highlight its dominant role compared to other sources within the drinking water reservoir catchment (it addresses pollution from one-quarter of the population of the monitored catchment area). The average effluent flow is 66 L.s^−1^ (SD 19; median 61; max 106; min 46).

Water quality was also monitored at 15 additional profiles along the River basin:

e) Four stabilization (biological) ponds (if) located downstream of the municipalities to further improve water quality. These ponds effectively reduce pollution; however, under certain conditions (e.g., during annual recovery cycles), they can re-release pollutants.

One of the monitored stabilization ponds is part of the additional purification stage for the large WWTP discharge. There are two series-connected ponds and outflow from the second one was monitored. The outflow from this stabilization pond consists only of treated water; therefore, it is not burdened by occasional stormwater overflows and is not diluted with water from the recipient. The average effluent flow is 28 L.s^− 1^ (SD 9.8; median 28; max 40; min 16).

The other three stabilization ponds assessed are located downstream of the small conventional WWTPs as additional purification stages. These stabilization ponds are built on a recipient that receives the treated wastewater. The recipient can be occasionally burdened by stormwater overflows. The average effluents flows are 1.4 L.s^− 1^ (SD 1.2; median 0.5; max 3.7; min 0.2).

f) Nine profiles located 1 km downstream of the pollution sources (either IAS, WWTP, or stabilization pond). These profiles were selected so as not to be affected by other sources of urban pollution discharges. The average flow rate was 1.8 L.s^−1^ (SD 1.9; median 0.8; max 7.6; min 0.1).
g) Two control profiles upstream of the pollution discharges, with the average flow rate of 1.7 L.s^−1^ (SD 1.3; median 1.6; max 3.9; min 0.2).

### Sampling & analyses

A total of 184 24-hour sampling campaigns were conducted across 46 sampling profiles within 19 municipalities. Each profile was monitored at least three times (and up to seven times) to capture seasonal dynamics. Samplings was primarily performed during spring, summer, and autumn periods; winter sampling was generally omitted due to technical challenges associated with automatic samplers freezing. To ensure a representative characterization of wastewater quality, each 24-hour campaign yielded one composite sample consisting of 96 individual increments, collected at 15-minute intervals. The campaigns were scheduled to ensure that flow rates were not affected by precipitation events. The flow rate was measured during each campaign using flow-rate measuring flumes (Thompson, Parshall). In locations without permanently installed flow-rate measuring profiles, a waterproof plywood board with a geometric weir (Thompson, Cipoletti) was used to derive flow rates. The flow rate was recorded at one-second intervals by Solinst Levellogger pressure probes, with atmospheric pressure correction provided by Solinst Barogger. Simultaneously, several (up to eight) manual flow-rate measurements were carried out during each 24-hour measurement period.

Standard chemical analyses were provided by the accredited laboratories of T. G. Masaryk Water Research Institute in Prague. A total of 92 PPCPs were monitored (listed in Supplementary Information (SI), Tab. S1), including non-steroidal anti-inflammatory drugs (NSAIDs), antibiotics, β-blockers, lipid regulators, X-ray contrast agents, various sweeteners, psychostimulants, etc. Monitored substances were selected based on the revision of Directive 98/83/EC (the Proposal for a Directive of the European Parliament and of the Council on the quality of water intended for human consumption). PPCPs analyses were carried out according to EPA method 1694 in the Laboratory of Vltava River Basin Authority, state enterprise. Substances were separated and detected by LC–MS/MS methods based on direct injection of the sample into a chromatograph. A 1290 ultra-high-performance liquid chromatograph (UHPLC) coupled with an Agilent 6495B Triple Quad Mass Spectrometer (MS/MS) of Agilent Technologies, Inc. (Santa Clara, CA, USA) was used.

In many cases, the analyzed concentrations were below the limit of quantification (LOQ). There are many procedures to deal with such situations and to evaluate data responsibly. We proceeded according to Article 5 (Calculation of Mean Values) in Commission Directive 2009/90/EC. Guidelines address the situation when the amounts of a physicochemical or chemical measurand is below the LOQ, the measurement results shall be set to half of the LOQ concerned for calculation of mean values. This results in censored values. The inclusion of censored values leads to biased estimates of summary statistics (as does the excluding of censored values and counting only measured values). In the case of a significant occurrence of censored values in the data set, it is necessary to use some other processing than classic.

We included PPCPs with at least 50% uncensored values in the GWF calculation; meaning those detected in at least half of the monitored cases. The procedure is based on the international standard ISO 5667-20:2008 (Water quality - Sampling - Part 20 Guidance on the use of sampling data for decision making - Compliance with thresholds and classification systems).

### Grey water footprint calculation

The GWF of a monitored system is determined by the substance with the highest GWF value, according to the Eq. (1):

1 $$\:GWF=max\left\{{GWF}_{1},{GWF}_{2},\:\dots\:{GWF}_{n}\:\right\}$$

The GWF for a particular substance is calculated according to the Eq. (2):

2 $$\:{GWF}_{i}=\:\frac{{L}_{i}}{{c}_{max,i\:}-\:{c}_{nat,i}}$$

where:

GWF_i_ grey water footprint of substance *i* [volume/time].

L_i_ discharge load of substance *i* [mass/time].

c_max, i_ maximum acceptable concentration of substance *i* in the aquatic environment [mass/volume].

c_nat, I_ natural concentration of substance *i* in the aquatic environment [mass/volume].

When evaluating the GWF, special attention must be paid to the concentration limits, as these strongly affect the final GWF value^27–29^. For synthetic substances that are not naturally found in ecosystems, we consider c_nat, i_ = 0^9^. Some difficulties arise when searching for the most relevant maximum acceptable concentrations (c_max_) of micropollutants. For the safe concentration in the aquatic environment, several terms are used in different risk assessment methodologies. These include *‘trigger value’*,* ‘threshold concentration’*,* ’critical concentration’*,* ’benchmark level’*, etc. The most frequently used endpoint in environmental risk assessment to summerize the overall hazard assessment is the predicted no-effect concentration (PNEC). The European Commission defines PNEC_i_ as the concentration of substance i, below which no adverse effects from environmental exposure are expected (Regulation EC 1907/2006). PNECs are usually derived using ecotoxicity testing data; obtained by selecting the most sensitive bioassay (representing the most sensitive trophic level) and applying an appropriate Assessment Factor (AF) that considers intra- and inter-laboratory data variability, biological variance, short- and long-term extrapolation, and extrapolation from laboratory to the field conditions.

For this study, we chose to use PNECs listed in the NORMAN ecotoxicology open-acess database (www.norman-network.com/nds/ecotox). These data are established upon based on pan-European expert consultations, and the values are periodically updated. The lowest PNEC derived for freshwater reflecting long-term exposure was used for the calculation. A comparison list of monitored PPCPs with their lowest PNECs published by different authors is given in SI (Tab. S2).

For organic and nutrient pollution, the natural and maximum-acceptable concentrations are set by the Czech Technical Standard ČSN 75 7221, which classifies surface water quality. Another possible source of these data is the methodology used for assessing the status of water bodies according to the Water Framework Directive (WFD) in the Czech Republic^30^. This methodology uses stricter limits and considers the specific characteristics of the assessed area (altitude, geological subsoil, and recipient size). We consider the values stated in the methodology^30^ more relevant for calculating the GWF in the watershed of the drinking water reservoir. For this study, the GWF was calculated using both sources of data to highlight the differences. The c_nat_ and c_max_ values used for the GWF calculation are given in Table 1.

**Table 1:** Natural and maximum acceptable concentrations used for the GWF calculation of organic and nutrient pollution.

	COD [mg·L^–1^]	*N*-NH_4_^+^ [mg·L^–1^]		*N*-NO_3_^−^ [mg·L^–1^]		*P*_tot_ [mg·L^–1^]	
c_nat_	15	0.2	0.06	2.5	1.7	0.05	0.035
c_max_	25	0.4	0.1	5	3.2	0.15	0.05
Source*	A	A	B	A	B	A	B

*Source A - Czech Technical Standard ČSN 75 7221. B - Methodology for assessing general physicochemical components of the ecological status of surface water bodies – category Rivers^30^.

### Environmental grey water footprint sustainability assessment

The relationship between the calculated GWF and the flow rate of the recipient was assessed, and the Water Pollution Level (WPL) was determined according to the Eq. (3):

3 $$\:WPL=\:\frac{GWF}{{WA}_{blue}}=\frac{GWF}{{R}_{act}}$$

where:

WPL water pollution level [-].

WA_blue_ blue water availability [volume/time].

R_act_ flow rate in recipient (run-off) [volume/time].

The WPL indicates the extend to which the watercourse’s assimilative capacity has been used to absorb pollution^31^. When WPL > 1.0, ambient water quality standards are violated. WPL < 1.0 indicates that there is sufficient water to dilute the pollutant load to meet the maximum acceptable concentrations^9^. This strict assessment is debated by some authors, who consider the uncertainties in determining the individual quantities used to calculate WPL^13,33^. Other authors incorporate uncertainties in the calculation of the GWF^33,34^.

## Results and discussion

### Raw wastewater

The GWF of wastewater entering WWTPs from sewage networks was assessed. Out of the 92 monitored PPCPs, only 7 were not detected at any location, during any campaign. Not detected substances are listed in SI, Tab. S3; descriptive statistics of all monitored parameters in SI, Tab. S4.

**Fig. 1:**
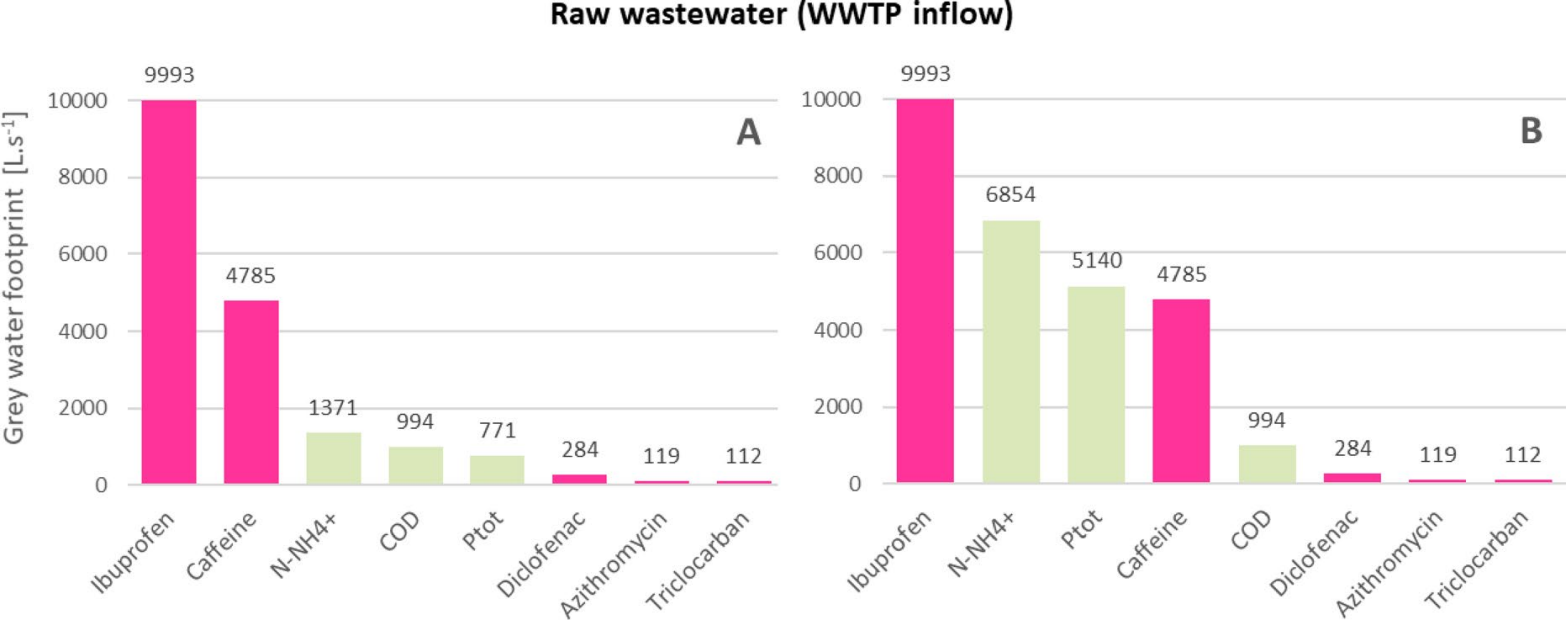
GWF of the most significant pollutants in raw wastewater (pink bars represent micropollutants (PPCPs); green bars represent macropollutants (organic and nutrient pollution). *Part A: c_max_ according to ČSN 75 7221; Part B: c_max_ according to^30^, as detailed in Table 1.

The highest GWF values were contributed by Ibuprofen and Caffeine (among PPCPs), as well as by nutrient and organic pollution. Previous studies on urban macropollution in the Czech Republic^12,29^ reported ammonium nitrogen as the decisive pollutant in raw wastewater. Substances with the most significant GWF values are shown in Fig. 1.

### Wastewater addressed via individual appropriate systems

Previous results^35^ demonstrate that municipalities with IAS impose the highest burden on the recipient water body. Only 16 out of the 92 PPCPs monitored were not detected in outflows from combined sewers where wastewater from IAS is discharged (overview in SI, Tab. S3, detailed in Tab. S4).

**Fig. 2:**
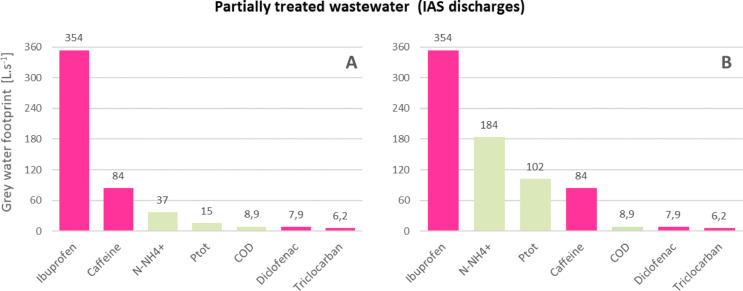
The GWF of the most significant pollutants discharged into recipients from sewerage systems with IAS. *Part A: c_max_ according to ČSN 75 7221; Part B: c_max_ according to^30^, as detailed in Table 1.

Seventy-six micropollutants were detected in IAS discharges, with Ibuprofen being the most decisive pollutant. The presence, and GWF significance of both, micro- and macro pollutants, (Fig. 2) correlate with those found in untreated wastewater (Fig. 1). This underscores the need for IAS to ensure the same level of environmental protection as secondary and tertiary treatments, as mandated by the recast Urban Wastewater Treatment Directive 2024/3019 (rUWWTD). These data confirm the importance of monitoring Ibuprofen and Caffeine as indicators of microcontaminants. A general strategy is needed to reduce the emissions of emerging contaminants from untreated or partially treated wastewater, among which Ibuprofen, Caffeine, Diclofenac pose the greatest risk^36^.

### Centrally treated wastewater (WWTP outflows)

None of the 16 substances listed in SI (Tab. S3) was detected in the WWTP outflows, at any location, during any campaigns. None of the monitored parabens (Methyl-, Ethyl-, Propyl-, Butylparaben) was detected, although they are commonly found in WWTP outflows in other regions^37,38^, which justifies their presence in aquatic natural environments^39^. The reason for this is likely the very low level of industrialization in the assessed area.

The removal efficiency of micropollutants varies considerably according to the technological processes used for water treatment^40–42^. Due to the diverse properties of micropollutants (e.g. hydrophobicity and biodegradability) and their low concentrations in urban wastewater, removal in conventional WWTPs with activated sludge is usually incomplete and variable. Removal generally refers to losses of the parent compound caused by various mechanisms of chemical and physical transformation, biodegradation, and sorption o solids^43,44^. The efficiency is influenced by the operational conditions, such as the organic load, redox conditions, sludge retention time, or temperature^45^. Micropollutants can also be removed by sorption on biological sludge^40^, while sorption on primary sludge is rather limited^46,47^. To evaluate the GWF of centrally treated wastewater, the monitored treatment systems have been divided into three groups, based on technology and size:

#### Outflows from small conventional WWTPs

**Fig. 3:**
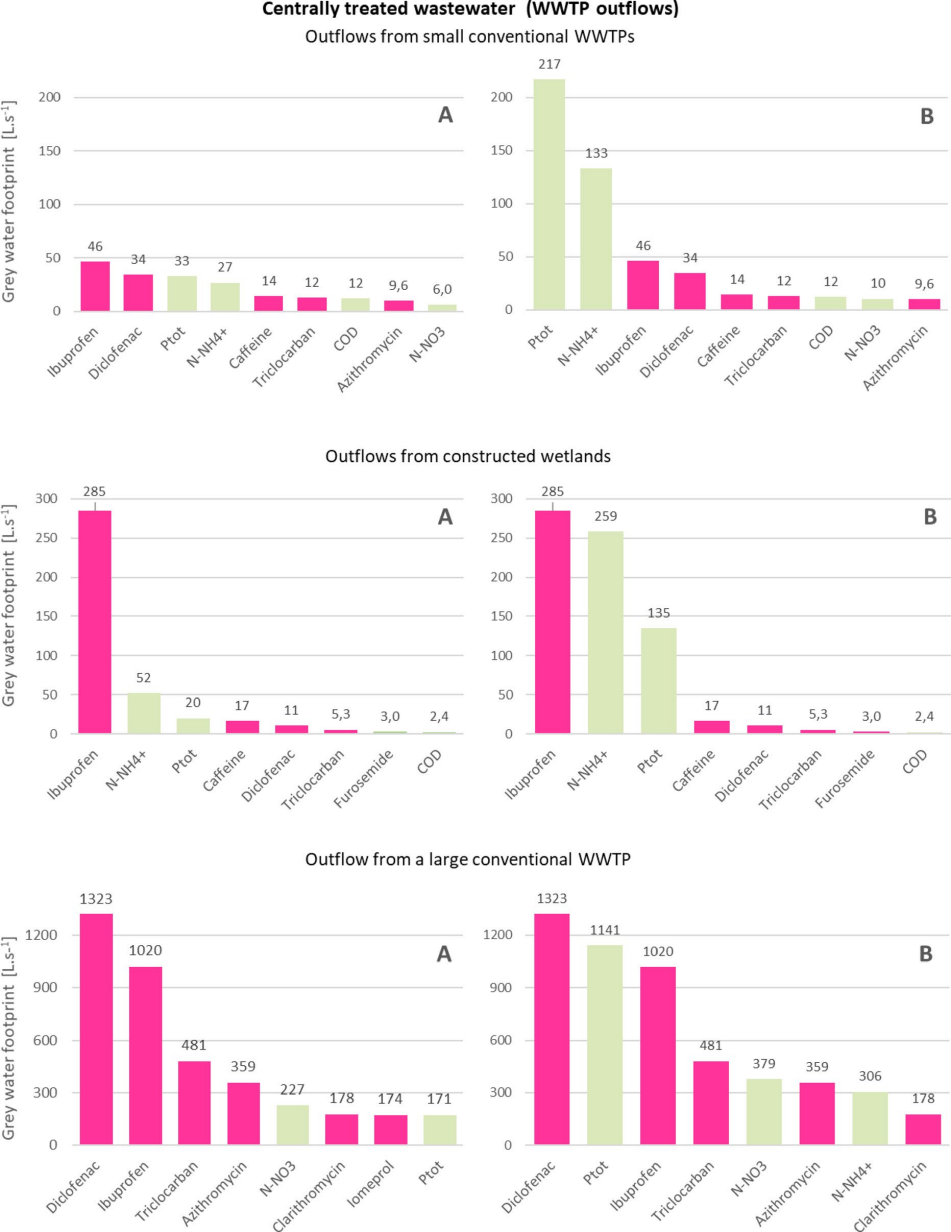
The GWF of the most significant pollutants discharged from various types and sizes of WWTPs. *Part A: c_max_ according to ČSN 75 7221; Part B: c_max_ according to^30^, as detailed in Table 1.

Eight small mechanical-biological WWTPs utilizing activated sludge was monitored. In terms of GWF, the most significant pollutant is Ibuprofen. However, under a stricter assessment of nutrient significance (Fig. 3, part B), the GWF is determined by total phosphorus. Other significant pollutants include Diclofenac (NSAID), Caffeine (stimulant), Triclocarban (an antibacterial agent used in soaps), and all monitored parameters of nutrient and organic pollution. From the group of antibiotics, Azithromycin, Clarithromycin, and Clindamycin contribute significantly.

#### Outflows from small unconventional WWTPs

Constructed wetlands (CWs) are unconventional, nature-based wastewater treatment systems. An overview of the most decisive pollutants discharged from CWs is shown in Fig. 3. As with small conventional WWTPs, Ibuprofen reaches the highest GWF, even under a more stringent assessment of nutrient significance. Ibuprofen removal occurs more easily in aerobic conditions^35^. A significant GWF is also attributed to ammonium nitrogen which is rarely present in outflows from well-operated conventional WWTPs. The presence of ammonium nitrogen indicates prevailing conditions with low dissolved oxygen concentration in the treatment beds.

#### Outflow from a large conventional WWTP

A large, well-equipped and well-operated mechanical-biological WWTP with low activation was evaluated separately. In this case, the GWF of the discharged pollution is dominated by Diclofenac, although Ibuprofen is also significant (both NSAIDs). Under a more stringent nutrient assessment, total phosphorus is highly relevant. An overview of the GWF significance is in Fig. 3. The presence of Triclocarban, the antibiotics Azithromycin and Clarithromycin, and nutrients shows a similar representation and proportion of pollutants as in the effluents from small conventional WWTPs.

The efficiency of micropollutant removal via biological wastewater treatment processes depends on the physicochemical properties of each contaminant, the treatment technology, the hydraulic retention time, the sludge age, the climatic conditions (e.g. wastewater dilution by rainfall, temperature, solar radiation)^45,48^, and the operational and technical conditions of the WWTP. Many studies report that Ibuprofen and Caffeine are among the micropollutants easily removed at WWTPs^49–52^. The mechanical-biological WWTPs monitored in this study remove Ibuprofen, Caffeine/Paraxanthine, Paracetamol, and Metformin with high and stable efficiency, although these remain present in the final effluents at trace concentrations. In general, Ibuprofen removal (> 95%) is among the highest of all pharmaceuticals, as also found in other studies^53^. However, despite its high removal efficiency, the GWF_Ibuprofen_ in WWTP discharges remains highly significant because its “safe” threshold limit for the aquatic environment is set very low for precautionary reasons.

Ibuprofen and Caffeine may be considered anthropogenic indicators of untreated wastewater discharges^54^. Ibuprofen can be toxic to various aquatic organisms, even at low concentrations, and as studies^55–57^ show, it can cause growth inhibition, reproductive problems, endocrine disruptions, and developmental abnormalities in fish and invertebrates. Although it does not bioaccumulate significantly, it can lead to chronic exposure and its residual presence in drinking water raises concerns about potential risks. Furthermore, transformation products released from WWTPs may be more toxic than the original compound, exacerbating the environmental contamination problem^58^.

Conversely, there are substances of a more stable nature that resist treatment processes. In the literature, Carbamazepine (anti-epileptics, used in the treatment of Alzheimer’s disease), Diclofenac (anti-inflammatory analgesics), Metoprolol (high blood pressure treatment), Clarithromycin (macrolide antibiotic, one of the most prescribed pharmaceuticals in human medicine), and Sulfamethoxazole (bacteriostatic sulfonamide antibiotic) are often mentioned as being resistant to conventional treatment^49,51,59–62^. This is confirmed by the fact that they are among the pharmaceuticals most frequently detected in effluent-impacted rivers^59,60^. Ecotoxicological studies of these pharmaceuticals do not yet indicate that they cause acute toxic effects in the environment at current concentrations^63,64^; however, the risk of their chronic effects requires increased attention^65,66^.

### Additional treatment in stabilization ponds

Stabilization ponds were constructed in the assessed basin as other nature-based systems for additional purification. As described in the *Monitoring* chapter, two types of stabilization pond configurations were assessed. One serves as a tertiary treatment stage for the large WWTP - there are two series-connected ponds, and outflow from the second one was monitored. The outflow (average flow rate of 28.0 L.s^− 1^) consists solely of treated water; thus, it is neither affected by occasional stormwater overflows nor diluted by the receiving water. The remaining three stabilization ponds are located on the receiving stream downstream of small conventional WWTPs. Unlike the former, these ponds may be occasionally burdened by stormwater overflows. The average effluents flow rate from these ponds was 1.4 L.s^− 1^.

Thirty-three substances that were not detected in the water discharged from the stabilization ponds are listed in SI, Tab. S3; descriptive statistics of all monitored parameters in SI, Tab. S4.

**Fig. 4:**
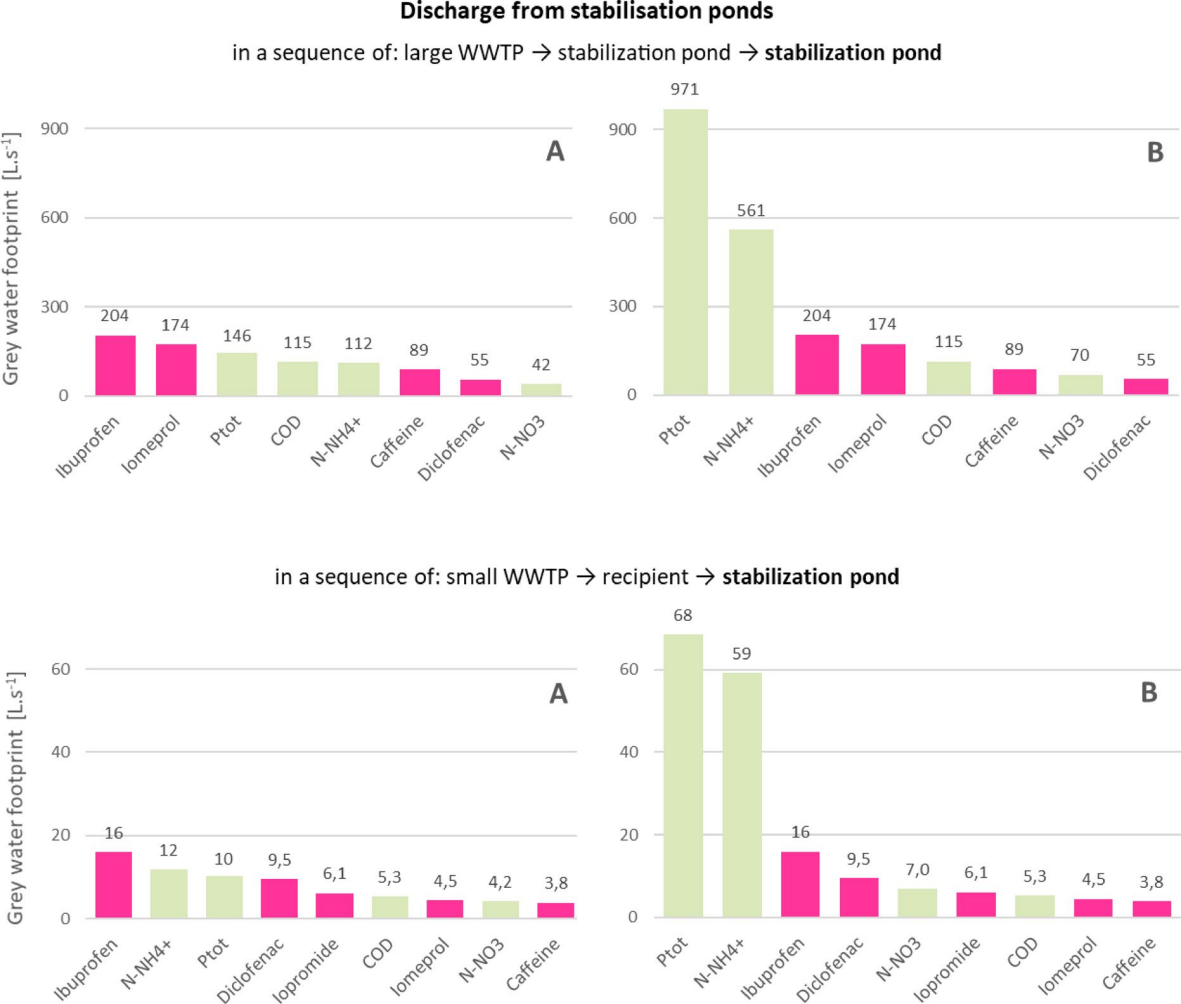
The GWF of the most significant pollutants discharged from stabilisation ponds. *Part A: c_max_ according to ČSN 75 7221; Part B: c_max_ according to^30^, as detailed in Table 1.

The spectrum of the most significant pollutants is similar in the outflows from both types of stabilization ponds configurations. They differ only in the GWF value because the outflow rates from the stabilization ponds downstream of the small WWTPs are lower. The most significant pollutant is Ibuprofen, while under a more stringent assessment of nutrient significance, total phosphorus and ammonium nitrogen are dominant. Other significant micropollutants include Diclofenac, Iopromid and Iomeprol (X-ray agents), and Caffeine. All monitored parameters of nutrient and organic pollution are among the significant pollutants, as detailed in Figs. 4.

### Recipients

In the receiving waters monitored 1 km downstream from the assessed pollution sources, a total of 25 monitored substances were not detected during any of the campaigns (the list is given in the SI, Tab. S3; more detailed in Table 4).

Fifty-four substances had a GWF value lower than 0.5 L.s^− 1^. The remaining 11 PPCPs (Ibuprofen, Caffeine, Diclofenac, Iopromide, Iomeprol, Sertraline, Clarithromycin, Clindamycin, Oxypurinol, Furosemide, Azithromycin) and all four monitored organic and nutrient pollution parameters have GWF values significant enough to be mentioned. To better understand the type of pollution causing the greatest burden in the recipient, the data related to the recipient were divided based on the different treatments adopted by the municipalities. Three scenarios were evaluated:

#### Recipients downstream of IAS discharges

GWF in recipients downstream discharges from municipalities addressing the pollution via IAS; fifteen 24-h measurements at 5 different localities were carried out; the average flow in recipients was 1.2 L.s^− 1^ (SD 1.6; median 0.4; max 5.5).

**Fig. 5:**
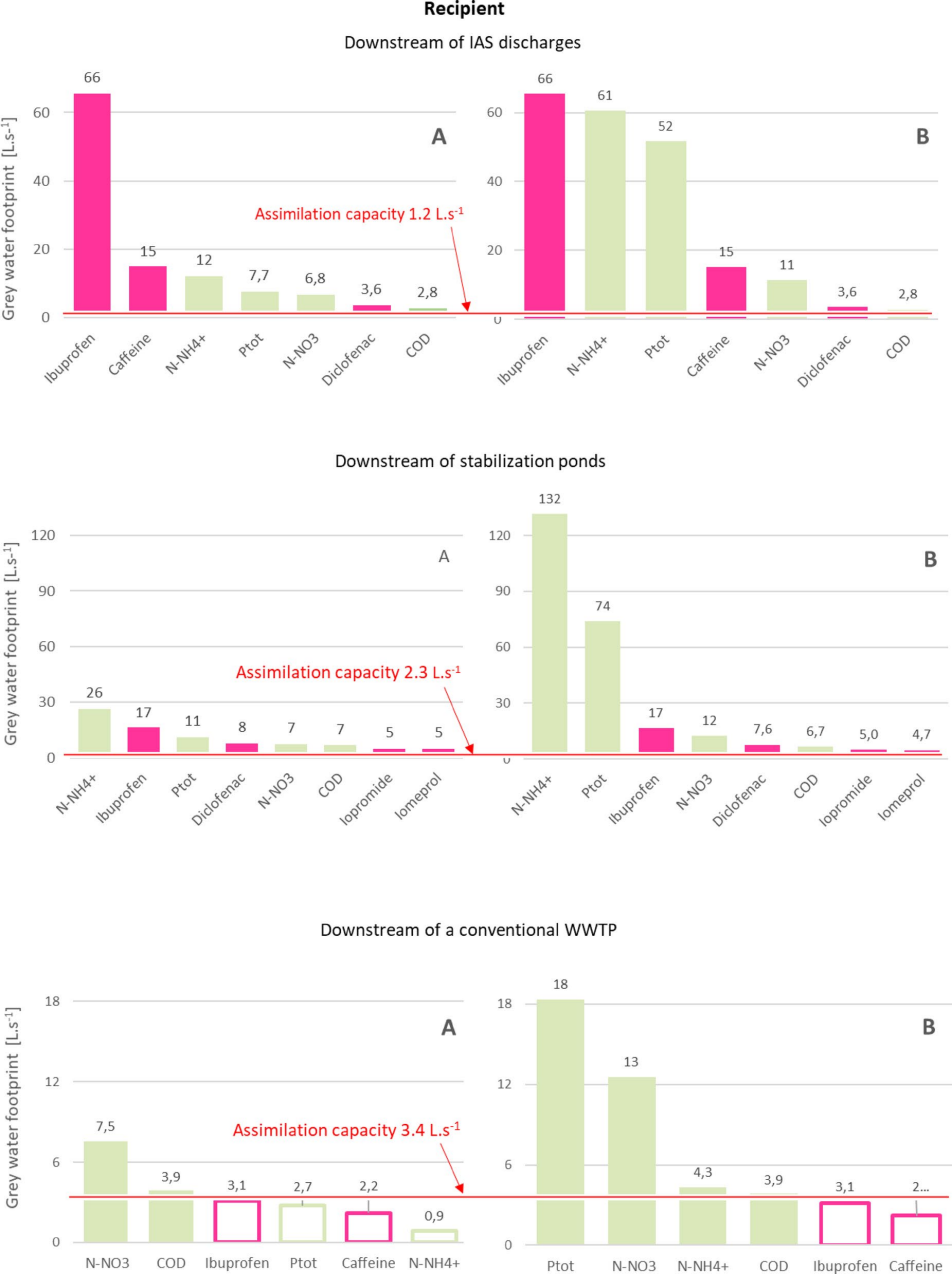
The GWF of the most significant pollutants in recipients downstream of pollution sources. The red line indicates the flow rate in the watercourse (i.e. blue water availability for the pollution dilution). *Part A: c_max_ according to ČSN 75 7221; Part B: c_max_ according to^30^, as detailed in Table 1.

The GWF in the watercourse is determined by Ibuprofen (GWF_Ibuprofen_ = 66 L.s^− 1^, SD 85). Although it is easily to remove, in the absence of central wastewater treatment, it contributes significantly to the burden on the recipient. Figure 5 shows all pollutants whose water pollution level exceeds 1 (WPL > 1), meaning that the recipient flow is insufficient to dilute the pollution to the level of the agreed standards. WPL_Ibuprofen_ = 55, meaning the water needed to dilute the pollution exceeds the recipients flow rate by 55 times. The other two micropollutants which burden the recipient are Caffeine and Diclofenac. Considering macropollutants, all four monitored parameters exceed the assimilation capacity of the watercourse, meaning they are above the limits of sustainability.

#### Recipients downstream of stabilization ponds

GWF in the recipients downstream stabilization ponds; nine 24-h measurements at 3 different localities were carried out; the average flow in the recipients was 2.3 L.s^− 1^ (SD 2.1; median 1.5; max 7.6).

The GWF in the watercourse is determined by ammonium nitrogen. This is explained by the low-oxygen conditions in stabilization ponds where digestion often occurs. The GWF_N−NH4+_ exceeds the watercourse’s assimilation capacity 11 times (WPL = 11), and even 57 times (WPL = 57) when the stricter nutrient assessment limits are applied. All pollutants exceeding the sustainability limit of the watercourse (WPL > 1) are shown in Fig. 5. From the entire spectrum of 92 monitored PPCPs, four micropollutants exceed the watercourse’s assimilation capacity: Ibuprofen, Diclofenac, and the X-ray contrast agents Iopromide and Iomeprol. As a result of outpatient diagnostics, contrast agents were not typically restricted to specific hotspots (i.e., district hospital) but observed in all monitored profiles more or less evenly.

#### Recipients downstream of a conventional WWTP

GWF in a recipient downstream a small mechanical-biological WWTP; three 24-h measurements at 1 monitored locality; the average flow in the recipient was 3.4 L.s^− 1^ (SD 1.4; median 3.4; max 5.2).

Only one profile was monitored in this scenario, as other locations were not evaluated as suitable (the basic condition being a stream segment hundreds of meters long downstream of the WWTP discharge, unaffected by any other point source of pollution). The GWF in the watercourse downstream of the small WWTP was determined by nutrient pollution; specifically, due to the oxygen ratio in this case, by nitrite nitrogen (GWF = 7.5 L.s^− 1^ (SD 9.0) exceeding the watercourse’s assimilation capacity by more than twice, WPL = 2.2). It is difficult to determine the extent to which the nitrites represent residual pollution from the WWTP or originate from diffuse sources - runoff from fields. In the case of the stricter nutrient assessment limits, the most significant pollutant is total phosphorus (GWF = 18 L.s^− 1^ (SD 6.1); WPL = 5.3). In both cases, organic pollution is also significant (GWF = 3.9 L.s^− 1^ (SD 1.7); WPL = 1.1), as shown in Fig. 5. None of the 92 monitored micropollutants has the GWF higher than the sustainability limit given by the blue water availability in the watercourse (i.e. WPL > 1).

### CONTROL

As control profiles, not affected by any central urban wastewater discharges, two watercourses were monitored, about ten meters upstream of WWTP discharges. Water quality was monitored during three sampling campaigns at each profile. In total, 79 PPCPs (out of 92 monitored) were not detected (see SI, Tab. S3). The following 13 PPCPs were detected at least once in any of the control profiles: Metformin, DEET and Paracetamol were detected in all cases. Caffeine, Saccharin, Paraxanthine, Ibuprofen-carboxy and Gabapentin were detected in half of the cases. Acesulfame and Cotinine were detected in a minority of cases. Ibuprofen, Sertraline and 4-Formylaminoantipyrine were detected once. Detailed descriptive statistics of the measured concentrations are provided in SI (Tab. S4).

**Fig. 6:**
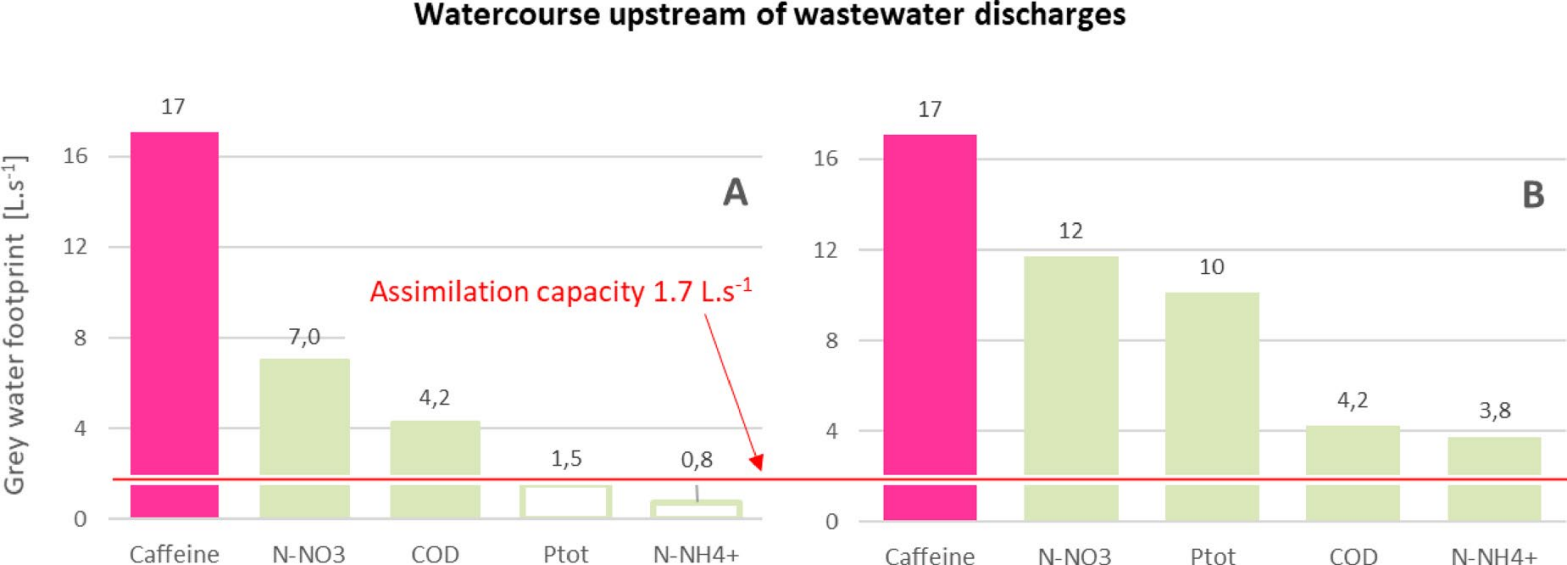
The GWF of the most significant pollutants in the control profile (watercourse upstream of wastewater discharge). All pollutants with WPL > 1 are shown as full bars. The red line indicates the blue water availability in the watercourse. *Part A: c_max_ according to ČSN 75 7221; Part B: c_max_ according to^30^, as detailed in Table 1.

Caffeine was determined as the pollutant with the highest GWF (WPL = 10.0), despite being detected only in half of the cases. Although Caffeine is less toxic than Ibuprofen, it can cause behavioral changes in aquatic organisms^67–69^. However, its residual presence in drinking water is a minor issue compared to other contaminants. Regarding the macropolutants, the flow rate in the watercourse is insufficient to dilute pollution by nitrates (WPL = 4.1) and COD (WPL = 2.5). In the case of stricter nutrient limit settings (B), the WPL is exceeded by all monitored parameters of nutrient and organic pollution, as shown in Fig. 6. The average flow rate in the control watercourse was 1.7 L.s^− 1^ (SD 1.3; median 1.6; max 3.9).

Given that the control measurements are located upstream the WWTP discharge, but still below the municipality level, it can be concluded that the detected Caffeine pollution is likely caused by seepages from cesspits and septic tanks. Significant nitrate pollution may be caused by runoff from surrounding fields (as the Želivka River basin is in an agricultural landscape) and by seepage from livestock sites. Organic pollution can be attributed to both agricultural production and seepage from cesspits and septic tanks.

## Sensitivity analysis

The derivation of PNEC for aquatic systems is based on laboratory data from standardized tests, covering organisms across three key trophic levels: primary producers (algae), primary consumers (invertebrates), and secondary consumers (fish)^70^). The PNEC is obtained by identifying the most sensitive bioassay, representing the most vulnerable trophic level. Due to inherent uncertainties, applying a conservative Assessment Factor (AF = 1000, 100 or 50) to the ecotoxicological data can lead to substantial variation in PNECs^18,71^. Inconsistent AF selection and uneven treatment of acute and chronic data may result in PNECs varying by more than three orders of magnitude^72^. Given the GWF strong dependance variable values, a sensitivity analysis was performed to quantify their influence on final GWF results.

Relevant literature on micropolutants PNECs was reviewed for comparison^52,63,70,73–80^, including the periodically updated NORMAN Ecotoxicology Database. PNECs from different sources were compared (see SI, Tab. S2); and the lowest value for each pollutant was selected to recalculate GWF across all monitored profiles. The comparative analysis, according to NORMAN ecotoxicology data, yield the following results:

### Raw WW (WWTP inflows)

GWF at the WWTP inflows is caused by Ibuprofen (GWF_Ibuprofen_ = 9,993 L.s^− 1^ (SD 22,579; median 1,055; max 88,350)). When the lowest PNEC values are considered, Paraxanthine becomes dominant (GWF_Paraxantine_ = 41,128 L.s^− 1^ (SD 97,833; median 3,780; max 483,352)). Other significant contributors to GWF include Diclofenac (14,191 L.s^− 1^ (SD 32,173; median 1,624; max 128,684) and Ibuprofen 10,993 L.s^− 1^ (SD 34,837; median 1,162; max 97,185), followed by DEET, Caffeine, and Metformin, whose GWF were approximately 5 times lower than that of Paraxanthine. Remaining monitored micropollutants exhibit GWFs one or more orders of magnitude lower.

### Partially treated WW (discharges from IAS)

GWF in sewer discharges from IAS is dominated by Ibuprofen (GWF_Ibuprofen_ = 354 L.s^− 1^ (SD 331; median 258; max 1,842)). When the lowest PNEC values are considered, Paraxanthine becomes the primary contributor (GWF_Paraxantine_ = 568 L.s^− 1^ (SD 568; median 477; max 1,267)). Paraxantine, a metabolite of Caffeine, exhibits similar stimulatory effects as Caffeine and approximately 80% of ingested Caffeine is converted to Paraxanthine in the human body. Other important contributors to GWF, like raw wastewater, include Diclofenac (395 L.s^− 1^; SD 312; median 374; max 1,299) and Ibuprofen (390 L.s^− 1^; SD 364; median 284; max 2,026). GWF values of other pollutants were one or more orders of magnitude lower, except for DEET, which was approximately half an order of magnitude lower than the aforementioned compounds.

### Centrally treated WW (WWTP outflows)

GWF at the WWTP outflows is caused equally by Diclofenac (212 L.s^− 1^ (SD 479; median 19; max 1,912)) or Ibuprofen (211 L.s^− 1^ (SD 417; median 18; max 3,424)). Considering the lowest PNECs, Diclofenac remains the predominat (10,590 L.s^− 1^; SD 23,926; median 941; max 95,593); both overall and across all monitored treatment types and plant sizes.

### Additionally treated WW (outflows from stabilisation ponds)

GWF at stabilisation pond outflows is primarily caused by Ibuprofen (204 L.s^− 1^ (SD 130; median 197; max 367)), and 16 L.s^− 1^ (SD 15.7; median 8.1; max 54), depending on pond connection sequence, as shown in Fig. 4. Diclofenac dominates when considering the lowest PNECs, both for ponds treating large WWTP effluent (2,759 L.s^− 1^ (SD 1,484; median 3,678; max 3,934)), and for ponds providing additional treatment of small WWTP outflows (474 L.s^− 1^ (SD 481; median 291; max 1,604)). Other significant contributors include Telmisartan, Paraxantine, DEET, and Venlafaxine.

### Recipients (downstream central urban WW discharges)

GWF in recipients is driven by Ibuprofen downstream of IAS discharges (66 L.s^− 1^ (SD 84; median 15; max 298)), ammonium nitrogen downstream of stabilisation ponds (26 L.s^− 1^ (SD 73; median 0.4; max 232)), and nitrite nitrogen downstream of small conventional WWTPs (7.5 L.s^− 1^ (SD 7.9; median 2.0; max 18.7)). Considering the lowest PNECs, Atorvastatin dominates downstream of IAS discharges (298 L.s^− 1^ (SD 220; median 363; max 613)), despite being detected in only half of cases; Diclofenac is decisive downstream of stabilisation ponds (381 L.s^− 1^ (SD 373; median 283; max 1,152)); and DEET downstream of small conventional WWTP (295 L.s^− 1^ (SD 388; median 35; max 844)).

### Watercourses (upstream central urban WW discharges)

GWF in watercourses (set as control) is caused by Caffeine (17 L.s^− 1^ (SD 31; median 3.9; max 87)). Considering the lowest PNECs, DEET emerged as dominant pollutant (25 L.s^− 1^ (SD 36; median 12.3; max 105)). Other micropollutants exceeding the watercourse flow included Paraxantine (18 L.s^− 1^ (SD 19; median 12; max 53)) and Caffeine (17 L.s^− 1^ (SD 31; median 3.9; max 87)). Results of the sensitivity analysis are summarised in Table 2, which lists the decisive pollutant in each locations using (i) threshold limits from the Methodology (C_max_ for nutrients from the Czech National Standard; C_max_ for micropollutants as PNECs from the NORMAN database), (ii) stricter nutrients thresholds^30^, and (iii) stricter micropollutants thresholds (in SI, Tab. S2).

**Table 2:** Summary of sensitivity analysis results (micropollutants highlighted in italics, nutrients in bold).

Type of water assessed	More detailed description	Decisive polutant		
		When using threshold limits as described in Materials and methods	When using stricter threshold limits for nutrients	When using stricter threshold limits for micropollutants
Raw WW		*Ibuprofen*	*Ibuprofen*	*Paraxanthine*
Partially treated WW	Outflows from IAS	*Ibuprofen*	*Ibuprofen*	*Paraxanthine*
Centrally treated WW	Outflows from small conventional WWTPs	*Ibuprofen*	**Total Phosphorus**	*Diclofenac*
	Outflows from constructed wetlands	*Ibuprofen*	*Ibuprofen*	*Diclofenac*
	Outflow from a large conventional WWTP	*Diclofenac*	*Diclofenac*	*Diclofenac*
Additionally treated WW	Outflows from stabilisation ponds	*Ibuprofen*	**Total Phosphorus**	*Diclofenac*
Recipients downstream urban WW discharges	Downstream discharges from IAS	*Ibuprofen*	*Ibuprofen*	*Atorvastatin*
	Downstream stabilization ponds	**Ammonium**	**Ammonium**	*Diclofenac*
	Downstream small WWTP	**Nitrate**	**Total Phosphorus**	*DEET*
Recipients upstream urban WW discharges	Unaffected by WW discharge	*Caffeine*	*Caffeine*	*DEET*

## Limitation of the study and policy recommendation

Water Footprint Assessment Methodology (WFAM)^9^ assumes that individual pollutants do not interact, and the GWF is determined (at a particular location) by the substance with the highest value, without considering self-purification processes^9^, p.40]. Several authors have noted these limitations and proposed solutions^10,27^.

Paraiba et al.^81^ proposed a model to calculate the GWF of a pesticide mixture based on Finizio et al.^82^, using Loewe’s model of additivity (Concentration Addition, CA). According to Paraiba et al.^81^, the GWF depends on soil and pesticides physicochemical properties, applied doses, and the lowest concentration causing 50% effect (EC_50_) in the most sensitive aquatic organisms. The model assumes first-order pesticide degradation in soil and linear sorption and has been applied by others^83,84^.

A similar approach was adopted by Feng et al.^85^ who used human toxicity to calculate the GWF of metals. Another approach involves applying the Water Quality Index (WQI) from the Canadian Council of Ministers of the Environment, allowing flexibility in pollutant combinations^86–88^. An entirely different approach was proposed by Li et al.^89^, who determined the GWF in two phases. First, considering dilution and self-purification via a mass-balance model, and second, using fuzzy synthetic evaluation to account for uncertainties and determine the GWF “threshold”. Currently, no unified approach exists to address methodological issues in GWF calculations. This study follows WFAM^9^ based on current knowledge.

The role of self-purification processes, as for example pollutant decomposition into transformation products and their contribution to GWF, remains largely unexplored. Transformation products present two challenges: first, WWTPs may “produce” pollution by transforming one pollutant into another. The new substance will therefore apprear at higher concentrations in effluents than in influents (e.g., Oxypurinol/Allopurinol); second, discharged substances may transform further in the environment, potentially increasing toxicity.

While this study did not address these aspects, future research should identify substances that determine GWF values, considering both their dilution and that of their transformation products. This research is relevant for several reasons: (i) implementation of stricter environmental regulations and wastewater treatment standards (e.g., rUWWTD); (ii) changes in hydrological regimes due to climate change, reducing river dilution capacity and affecting water status, and (iii) reducing monitoring costs, as analyzing all pollutants, particularly emerging ones, is resource-intensive. Focusing on critical substances is necessary to determine GWF values efficiently.

Sensitivity analysis showed that using PNEC as “ambient water quality standards” directly affects GWF results and subsequent sustainability assessments. WFAM^9^ defines ambient water quality standards as ecological standards driven by policy objectives. Thus, GWF is suitable for achieving environmental goals, including the Sustainable Development Goals^90^ or the WFD^13^. The WFD defines a limited number of chemical status indicators, gradually expanded with new ones (review and update of Directive 2008/105/EC). GWF assessment, combined with WFAM sustainability analysis, can serve as a tool for evaluating chemical status under the WFD.

## Conclusions

This study provides a comprehensive assessment of 92 PPCPs, alongside conventional chemical parameters, in urban wastewater discharges across 19 municipalities within the watershed of the largest Czech drinking water reservoir. By combining extensive monitoring with the GWF methodology, it enables a quantitative comparison of the relative environmental burden of emerging and traditional pollutants in watercourses impacted by urban effluents.

The findings reveal that Ibuprofen and Diclofenac are the primary micropollutants (among PPCPs) in centrally treated wastewater, varying with technology and WWTP type, while Caffeine dominates upstream and in partially treated discharges. Conventional pollutants, particularly ammonium and nitrates, emerge as the main stressors approximately one kilometer downstream of effluent discharges, highlighting a paradox where nutrient pollution surpasses PPCP impacts, with only Ibuprofen remaining critical downstream in IAS discharges. This conclusion is limited to the monitored substances, as other contaminant classes, such as pesticides, were not included.

Hotspots, such as nursing homes, exhibit micropollutant concentrations an order of magnitude higher than other urban areas. Although many PPCPs were intermittently detected, eight substances (Metformin, Oxypurinol, Gabapentin, Acesulfame, Caffeine, Hydrochlorothiazide, DEET, and 4-Formylaminoantipyrine) were consistently present, identifying priority targets for monitoring. Despite relatively high concentrations, most were not significant pollutants from the GWF perspective, except Caffeine.

In watercourses not influenced by central wastewater discharge, Caffeine remains the most critical pollutant, as upsteam assimilation capacity is insufficient to dilute Caffeine, nitrates, and organic matter to safe levels.

By extending the GWF methodology with sensitivity analyses acounting PNEC variability, this study provides a transferable framework for evaluating and prioritizing contaminants across different pollutant categories. Paraxanthine, Diclofenac, and DEET were highlighted as critical under specific treatment scenarios, underscoring their relevance for ecological risk assessments.

This deep study on urban wastewater pollution provides insights for pollutant prioritization to improve water management and ecosystem protection. Future studies should update permissible concentrations of micropollutants in aquatic environments, considering synergistic or antagonistic interactions among micropolutants, and acknowledge that WWTP removal generally reduces parent compounds, which may transform into biologically active metabolites with unknown fate. Special attention should also be given to sludge reuse in agriculture as a potential source of diffuse contamination.

## Supplementary Information

Below is the link to the electronic supplementary material.

Supplementary Material 1

## Data Availability

The data supporting the findings of this study are available in Zenodo at [https://doi.org/10.5281/zenodo.18622885], under the terms of the Creative Commons Attribution 4.0 International license (CC BY 4.0).
